# Epigenetic Reader Bromodomain Containing Protein 2 Facilitates Pathological Cardiac Hypertrophy *via* Regulating the Expression of Citrate Cycle Genes

**DOI:** 10.3389/fphar.2022.887991

**Published:** 2022-05-25

**Authors:** Zhirong Lin, Zhenzhen Li, Zhen Guo, Yanjun Cao, Jingyan Li, Peiqing Liu, Zhuoming Li

**Affiliations:** ^1^ Department of Pharmacology and Toxicology, School of Pharmaceutical Sciences, National and Local United Engineering Lab of Druggability and New Drugs Evaluation, Guangdong Engineering Laboratory of Druggability and New Drug Evaluation, Guangdong Provincial Key Laboratory of New Drug Design and Evaluation, Sun Yat-sen University, Guangzhou, China; ^2^ International Institute for Translational Chinese Medicine, School of Pharmaceutical Sciences, Guangzhou University of Chinese Medicine, Guangzhou, China

**Keywords:** bromodomain containing protein 2 (BRD2), bromodomain and extra-terminal domain (BET) family, cardiac hypertrophy, cardiac metabolism, citrate cycle (TCA cycle)

## Abstract

The bromodomain and extra-terminal domain proteins (BETs) family serve as epigenetic “readers”, which recognize the acetylated histones and recruit transcriptional regulator complexes to chromatin, eventually regulating gene transcription. Accumulating evidences demonstrate that pan BET inhibitors (BETi) confer protection against pathological cardiac hypertrophy, a precursor progress for developing heart failure. However, the roles of BET family members, except BRD4, remain unknown in pathological cardiac hypertrophy. The present study identified BRD2 as a novel regulator in cardiac hypertrophy, with a distinct mechanism from BRD4. BRD2 expression was elevated in cardiac hypertrophy induced by β-adrenergic agonist isoprenaline (ISO) *in vivo* and *in vitro*. Overexpression of BRD2 upregulated the expression of hypertrophic biomarkers and increased cell surface area, whereas BRD2 knockdown restrained ISO-induced cardiomyocyte hypertrophy. *In vivo*, rats received intramyocardial injection of adeno-associated virus (AAV) encoding siBRD2 significantly reversed ISO-induced pathological cardiac hypertrophy, cardiac fibrosis, and cardiac function dysregulation. The bioinformatic analysis of whole-genome sequence data demonstrated that a majority of metabolic genes, in particular those involved in TCA cycle, were under regulation by BRD2. Real-time PCR results confirmed that the expressions of TCA cycle genes were upregulated by BRD2, but were downregulated by BRD2 silencing in ISO-treated cardiomyocytes. Results of mitochondrial oxygen consumption rate (OCR) and ATP production measurement demonstrated that BRD2 augmented cardiac metabolism during cardiac hypertrophy. In conclusion, the present study revealed that BRD2 could facilitate cardiac hypertrophy through upregulating TCA cycle genes. Strategies targeting inhibition of BRD2 might suggest therapeutic potential for pathological cardiac hypertrophy and heart failure.

## Introduction

Epigenetic modifications refer to dynamic and reversible changes in chromatin accessibility and post-translational modifications of histone tails, without altering nucleotide sequence (Prinjha et al., 2012). As the earliest and most in-depth studied epigenetic modification, histone acetylation plays important roles in chromatin remodeling and transcriptional regulation (Scher et al., 2007). Not only governed by the “writer” histone acetyltransferases and the “eraser” histone deacetylases, histone acetylation also relies on “readers”, which are acetyl-binding proteins recognizing the acetylated lysine in histones and recruiting transcriptional regulator complexes to chromatin, eventually regulating gene transcription (Wang et al., 2021). Nearly all known “epigenetic readers” contain bromodomains, which are about 110-amino-acid modules found in many chromatin-associated proteins (Scher et al., 2007). The bromodomain and extra-terminal domain (BET) family, consisting of four proteins (namely BRD2, BRD3, BRD4, and BRDT), is a subfamily of bromodomain-containing proteins (BRDs) designated because of the presence of two bromodomains along with an additional region of homology called ET domain (Gyuris et al., 2009). BET members are implicated in a majority of diseases, including human immunodeficiency virus infection (Boehm et al., 2013; Sharma et al., 2013), inflammatory diseases (Belkina et al., 2013), carcinogenesis (Mertz et al., 2011; Francisco et al., 2013), etc.

Pathological cardiac hypertrophy is characterized by enlargement of the heart and thickening of ventricular walls due to sustain and abnormal hemodynamic stress. Although it is initially an adaptive response to maintain cardiac function, prolonged cardiac hypertrophy exacerbates heart workload and facilitates the onset of heart failure (Tham et al., 2015). Maladaptive cardiac hypertrophy is also an independent predictor of adverse outcomes in patients with heart failure (Tham et al., 2015). Therefore, interventions to prevent pathological cardiac hypertrophy are in urgent need. Accumulating evidences demonstrate that BET inhibitors (BETi) exhibit protective effects against pathological cardiac hypertrophy. A pan-BETi, JQ1, ameliorates cardiomyocyte hypertrophy and left ventricular hypertrophy (LVH) induced by transverse aortic constriction (TAC) in mice (Spiltoir et al., 2013). Another BETi, apabetalone (RVX-208), improves the cardiovascular outcomes of patients with diabetes after acute coronary syndrome, suggesting its therapeutic potential for cardiovascular diseases (Ray et al., 2019). Among the four BET family members, BRD4 has been widely reported to be involved in regulation of cardiac hypertrophy (Van der Feen et al., 2019; Zhu et al., 2020; Li et al., 2021). However, the roles of other BET members in pathological cardiac hypertrophy remain unclear. Considering the broad effects of pan-BET, the possible involvement of other BET members could not be ignored. In our preliminary studies, we measured the mRNA levels of BET members in a cardiomyocyte hypertrophy model induced by β-adrenergic receptor activation. BRD2 was remarkably elevated at the transcriptional level in parallel with BRD4, suggesting that BRD2 is probably associated with the pathological progression of cardiac hypertrophy (Li et al., 2021).

BRD2 and BRD4 share similar structure, with about 80% identity at the amino acid level in human and mouse. The only difference is that BRD4 has a distinct longer C-terminal domain (CTD), which can facilitate transcription reactivation and elongation by recruiting and interacting with the positive-transcriptional elongation factor b (P-TEFb), while BRD2 does not have such structure (Belkina and Denis 2012; Taniguchi 2016). With its CTD function, BRD4 enhances the recruitment of P-TEFb, which phosphorylates Ser2 of RNA Pol II and promotes transcriptional pause release of Pol II (Spiltoir et al., 2013; Stratton and McKinsey 2015). However, it is still uncertain about the exact biological functions of BET family members without CTD, such as BRD2 and BRD3 (Belkina and Denis 2012). Some studies indicate that the main role of BRD2/3 might be to remodel the promoters and enhancers to activate the transcriptional initiation process, whereas BRD4 is primarily involved in transcription elongation (Kanno et al., 2014; Kim et al., 2021).

Considering the existent structural and functional differences between BRD2 and BRD4, it raises our interests to explore the role of BRD2 in cardiovascular diseases. In view of this, the present study attempted to determine the regulatory effect of BRD2 in pathological cardiac hypertrophy, and to explore the possible underlying mechanisms.

## Materials and Methods

### Primary Culture of Cardiomyocytes

Neonatal rat cardiomyocytes (NRCMs) were isolated from hearts of 1–3 day old Sprague–Dawley (SD) rats as described previously (Cai et al., 2020). The cardiomyocytes were cultured in Dulbecco’s modified Eagle’s medium (DMEM, Gibco, Grand Island, NY, United States) supplemented with 10% fetal bovine serum (FBS, Gibco) and 0.1 mM 5-bromodeoxyuridine (BrdU, Thermo Fisher Scientific, Rockford, IL, United States) and cultured at 37°C with 5% CO_2_. H9c2 cells purchased from the Cell Bank of the Chinese Academy of Sciences (Shanghai, China) were cultured under the same conditions but without BrdU. Cells were incubated with ISO (isoprenaline, MedChemExpress, NJ, United States, 10 μM final concentration) for 12 h to stimulate cardiomyocyte hypertrophy.

### Animal Experiments

The animal experiments were conducted following the Guide for the Care and Use of Laboratory Animals (NIH Publication No. 85-23, revised 1996) and were approved by the Research Ethics Committee of Sun Yat-Sen University. Sprague–Dawley (SD) rats (male, 180–220 g, SPF grade, Certification No. 1100112011043685) were purchased from and housed in the Experimental Animal Center of Sun Yat-Sen University (Guangzhou, China). Rats were housed in a temperature-controlled room (21–23°C) with a 12 h daylight/dark cycles and had free access to chow and water. A total of 60 rats were randomly divided into four groups, namely NS, NS+ISO, AAV-siBRD2, AAV-siBRD2+ISO group. For AAV affection, adeno-associated virus encoding siBRD2 (AAV-siBRD2, 1012 particles) or negative control (AAV-siNC, 1012 particles) were offered by intramyocardial injection, followed by subcutaneous injection of ISO (1.2 mg/kg/d) or an equal volume of normal saline (NS) for 7 days.

### Echocardiography and Histological Analysis

Rats were anaesthetized with 3% (v/v) isoflurane, and two-dimensional-guided M-mode echocardiography was performed with Technos MPX ultrasound system (Esaote, Genoa, Italy) as described in our previous reports (Braverman et al., 2013). Cardiac function indexes, including the ejection fraction (EF) and fractional shortening (FS) were measured. Afterwards, all animals were sacrificed. The hearts were then quickly removed, weighed and then used for other measurements. The upper portion of hearts were fixed in 4% paraformaldehyde, and then embedded in paraffin for morphometric measurement.

### Small Interference RNA and Plasmid Transfection

siRNA (small interfering RNA) against BRD2 (siBRD2) and negative control (NC) were obtained from GenePharma (Shanghai, China). Plasmid that expresses BRD2 was purchased from Sangon Biotech (Shanghai, China). Cells were transfected using Lipofectamine 2000 (Invitrogen, Carlsbad, CA, United States) with Opti-MEM^®^ I Reduced-Serum Medium (Gibco) following the manufacturer’s instructions. Cells were incubated with mixture for 5 h and replaced with normal culture media. The expression of mRNA and protein were measured after 48 h by qRT-PCR or Western blot analysis.

### Western Blot

Cells or myocardial tissue were washed for 3 times with cold PBS, following by extraction with RIPA lysis buffer (Beyotime, Nantong, Jiangsu, China) supplemented with protease inhibitors and phosphatase inhibitors (Bimake, Houston, TX, United States) on ice. Cell lysates were obtained by centrifugation at 12,000 g for 15 min at 4°C. After quantification using BCA Protein Assay Kit (Thermo Fisher Scientific), 30 μg of the lysates were boiled for 5 min in loading buffer at 90°C and subsequently separated in SDS-PAGE. The proteins were transferred onto polyvinylidene difluoride (PVDF) membranes and were then incubated by primary antibodies for 16 h at 4°C, followed by 1 h of secondary antibodies incubation at room temperature. The signals of protein level were visualized by Image Quant LAS 4000 mini (GE Healthcare, Waukesha, WI, United States). The intensities of the blots were quantified by the Quantity One (Bio-Rad, California, United States) software. α-tubulin or GAPDH (glyceraldehyde-3-phosphate dehydrogenase) were used as internal references.

Antibodies against BRD2 (bromodomain containing 2, Cat# A302-583A-M) were from Bethyl; anti-α-tubulin (Cat# 66031-1-IG) and GAPDH (Cat# 60004-1-IG) were obtained from Proteintech; anti-ANF (atrial natriuretic factor, Cat# PAA225Ra02) was bought from Cloud-Clone Corp. Normal HRP-conjugated secondary antibodies were obtained from cell Signaling Technology. Each antibody was used at the recommended dilution.

### RNA Extraction and RT-PCR Quantification

Total RNA was isolated from NRCMs or myocardial tissue by TRIzol Reagent (Takara Biotechnology, Dalian, China) according to the manufacturer’s instructions. 1–5 μg of total RNA was reversed and transcripted into first-strand cDNA using RevertAid First Strand cDNA Synthesis Kit (Thermo Fisher Scientific). Then, 1 μl cDNA, together with 1 μl of primer, 3 μl DEPC water and 5 μl SYBR Green Realtime PCR Master Mix (Toyobo Life Science, Osaka, Japan), were infused into one well of 96-well white plate and undergone relative quantitative RT-PCR analysis using LightCycler480 II (Roche, Basel, Switzerland). Data were normalized to β-Actin. The relative expression level was determined by 2^−ΔΔCt^ method. The following primers are listed in Supplementary Table S1 and synthesized by Sangon Biotech.

### Measurement of Cell Surface Area

Cardiomyocytes seeded in 48-well plates were washed with PBS and fixed in 4% paraformaldehyde for 15 min. Cells were then incubated in 0.03% Triton X-100 for 15 min. After incubation with 0.1% rhodamine phalloidin (Invitrogen) for 1h, the cells were washed with PBS and further stained with DAPI (Cell Signaling Technology, Danvers, MA, United States) for 10 min. All treatments were conducted at room temperature. The cells were then assessed with in High Content Screening System (Thermo Fisher Scientific), and 25 randomly selected fields were chosen for analysis by built-in image analysis software.

### RNA Sequence Analysis

Total RNAs of NRCMs were extracted as previous described and 10 μg total RNA of each sample was diluted in sterile and RNase-free water. Total 12 samples (3 duplicates for each group) were analyzed by BGI·Tech (Shenzhen, China). The mRNA was firstly enriched by magnetic beads bound with Oligo dT and then fragmented under high temperature conditions. The fragmented mRNA was used as template to synthesize one-strand cDNA, and then configure a two-strand synthesis reaction system to synthesize two-strand cDNA. After purification, end repairment, addition of base “A” to the 3’end of the cDNA and adapter connection, the product was finally amplified by PCR and sequenced. Bowtie2 software was used to map the clean reads to the reference genome (GCF_000001895.5_Rnor_6.0) (Langmead and Salzberg 2012). Gene expression levels of each sample were quantified by RPKM (Reads Per Kilobase per Million mapped reads) (Li and Dewey 2011). Totally 2371 genes overlapped in “BRD2-vs-CON” and “siBRD2+ISO-vs-ISO” group were used for GSEA analysis (Subramanian et al., 2005). Differential gene expression was assessed with DESeq2 R package, genes with an adjusted *P* value (*Q* value) ≤ 0.05 were considered to be differentially expressed (Love et al., 2014). The raw data have been deposited in the SRA database of NCBI (SRA accession: PRJNA830755).

### Measurement of Mitochondrial Oxygen Consumption Rate

OCR measurement was performed using an XF96 Extracellular Flux Analyzer (Seahorse Bioscience, North Billerica, MA, United States) with Seahorse XFp Cell Mito Stress Test Kit (Seahorse Bioscience) as described previously (Ding et al., 2021). Briefly, H9c2 cells were seeded into Seahorse XF96 microplates in a density of 2×10^4^ cells/well. For detection, the normal medium of the cells was replaced with XF assay medium containing 1 mM pyruvate, 2 mM glutamine and 10 mM glucose, and then incubated in 37°C/non-CO_2_ incubator for 1 h. The sensor cartridge was loaded with various compounds as follows: oligomycin (10 μM of final concentration), carbonyl cyanide 4-(trifluoromethoxy) phenylhydrazone (FCCP, 20 μM of final concentration) and rotenone/antimycin A (5 μM of final concentration), and then calibrated in the analyzer. The protein content quantified by BCA protein assay kit (Thermo Fisher Scientific) in each well was used for normalization. The data were analyzed with Wave software, and respiratory parameters were quantified. Every group was repeated with six duplicates.

### Measurement of Intracellular ATP Levels

ATP in the cell lysates was measured using a luciferase-based assay according to the manufacturer’s instruments (Beyotime). Data were normalized to total protein concentration of the cell lysate as determined by BCA protein assay kit (Thermo Fisher Scientific).

### Statistical Analyses

Data were expressed as average ± standard error of mean (SEM) of at least four independent experiments. Unpaired *t* test was used to determine differences between two groups. One-way ANOVA with Bonferroni post hoc test was used to determine differences among multiple groups. *p* < 0.05 was regarded to be statistically significant.

## Results

### Bromodomain Containing Protein 2 Expression was Elevated in ISO-Induced Cardiac Hypertrophy

**FIGURE 1:**
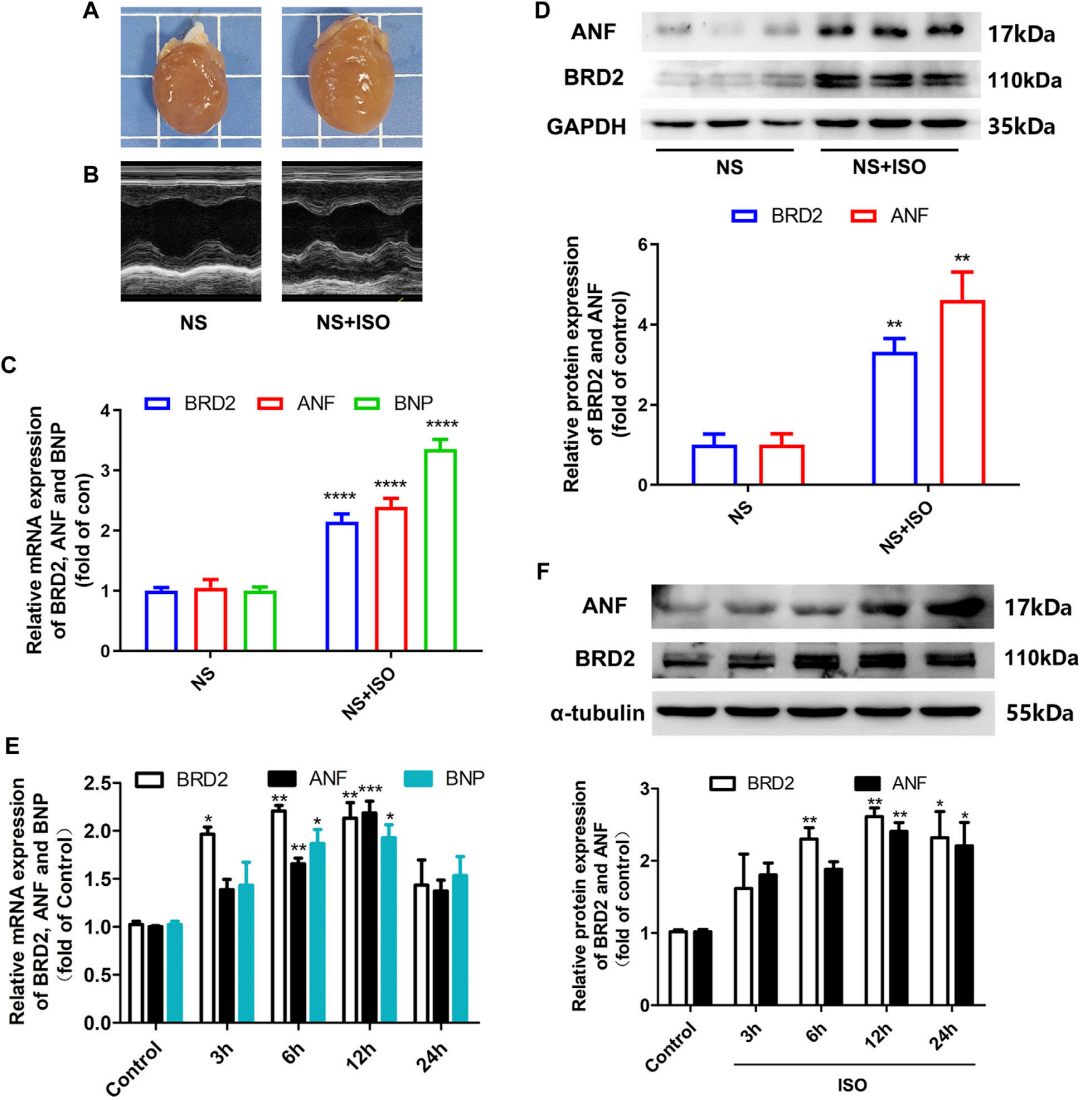
BRD2 expression was elevated in ISO-induced cardiac hypertrophy. Hypertrophic changes of left ventricle in ISO-treated rats were observed by **(A)** gross morphologic examination and **(B)** echocardiography. **(C)** The mRNA and **(D)** protein expression of BRD2, ANF and BNP in ISO-treated rats. **(E)** The mRNA and **(F)** protein expression of BRD2, ANF and BNP after treatment of 10 μM ISO for indicated times in cardiomyocytes. The data were presented as mean ± SEM, *p < 0.05, **p < 0.01, ***p < 0.001 *vs.* control, ****p < 0.0001 *vs.* control. *n* = 4.

Activation of β-adrenergic receptor induces calcium overload and oxygen free radical damage to cardiomyocytes, finally inducing cardiac hypertrophy (Grimm and Brown, 2010). In the present study, the classic β-adrenergic agonist isoprenaline (ISO) was used to stimulate cardiac hypertrophy. *In vivo*, 1.2 mg/kg/day of ISO was subcutaneously injected to SD rats for 7 days to induce cardiac hypertrophy. As shown in Figures 1A–D, these ISO-treated rats exhibited enlargement of heart size, thickening of left ventricular wall recorded by echocardiography, increased mRNA and protein expressions of hypertrophic biomarkers atrial natriuretic factor (ANF) and brain natriuretic peptide (BNP). In these ISO-induced cardiac hypertrophic rats, BRD2 expression was upregulated at both transcriptional and translational levels (Figures 1C,D). *In vitro*, NRCMs were incubated with 10 μM ISO to induce cardiomyocyte hypertrophy. Consistent with the *in vivo* observations, the mRNA and protein expression of BRD2 were elevated by ISO in a time-dependent manner, in parallel with the increase of ANF and BNP expressions (Figures 1E,F). These results thus imply the possible involvement of BRD2 in ISO-induced pathological cardiac hypertrophy.

### Bromodomain Containing Protein 2 Facilitated Cardiomyocyte Hypertrophy

**FIGURE 2:**
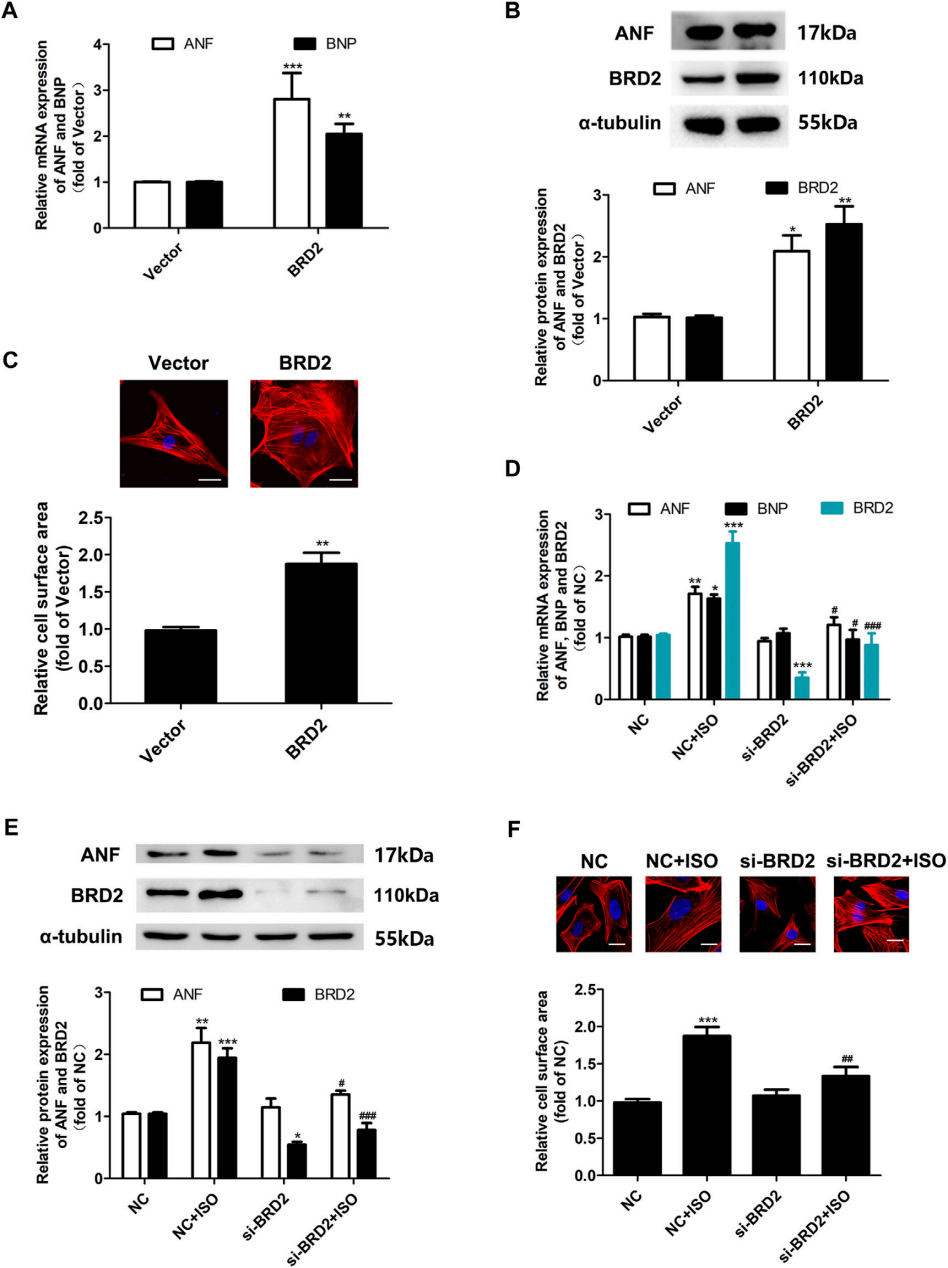
The effects of BRD2 in ISO-induced cardiomyocyte hypertrophy. **(A)** The mRNA level and **(B)** protein expression of BRD2, ANF and BNP, and **(C)** cell surface area measured by rhodamine-phalloidin staining, in cardiomyocytes transfected with BRD2 plasmid or blank vector for 48 h. Scale bar: 20 μm. **(D)** The mRNA level and **(E)** protein expression of BRD2, ANF and BNP, and **(F)** cell surface area measured by rhodamine-phalloidin staining, in cardiomyocytes transfected with siRNA targeting BRD2 (siBRD2) or negative control (NC) for 48 h, and further incubated with 10 μM ISO or DMSO for 24 h. The data are presented as mean ± SEM, *p < 0.05, **p < 0.01, ***p < 0.001 *vs.* control. ^#^ *p* < 0.05, ^##^ *p* < 0.01, ^###^ *p* < 0.001 *vs.* ISO. *n* = 4.

To investigate the exact role of BRD2 in cardiomyocyte hypertrophy, BRD2 was overexpressed by plasmid transfection, or knocked down by small interfering RNA (siBRD2, Supplementary Figure S1) in NRCMs. Overexpression of BRD2 elevated the mRNA and protein expression of hypertrophic biomarkers, and increased the cell surface area as indicated by rhodamine-phalloidin staining, suggesting that BRD2 could facilitate cardiomyocyte hypertrophy (Figures 2A–C). On the contrary, BRD2 knockdown restrained ISO-induced upregulation of ANF and BNP (Figures 2D,E), as well as expansion of cell surface area (Figure 2F). Taken together, these results suggest that BRD2 exerts pro-hypertrophic effects in cardiomyocytes, and that repression of BRD2 might help to ameliorate cardiomyocyte hypertrophy.

### Bromodomain Containing Protein 2 Knockdown Protected Sprague–Dawley Rats Against ISO-Induced Cardiac Hypertrophy

**FIGURE 3:**
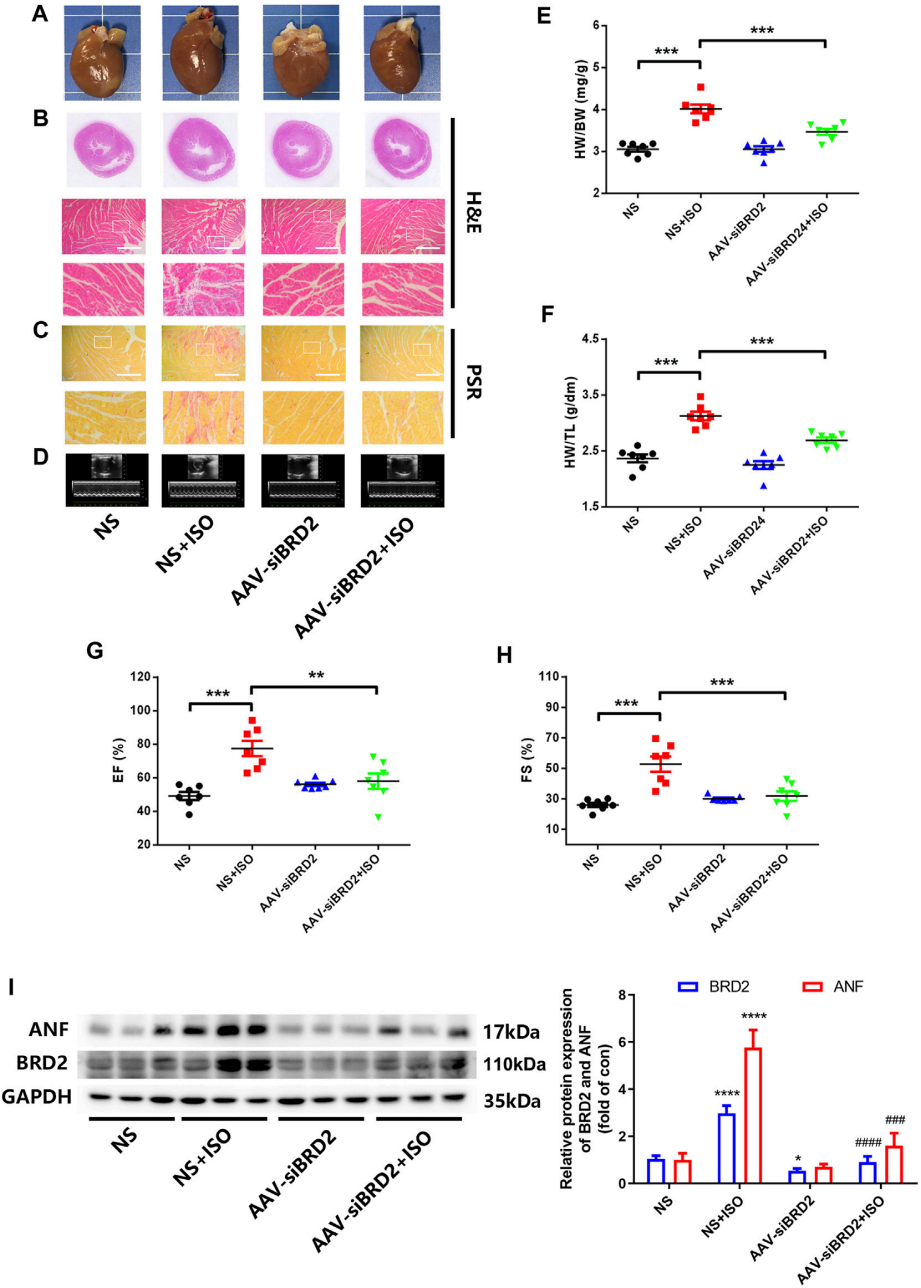
BRD2 knockdown protected SD rats from ISO-induced cardiac hypertrophy. SD rats received intramyocardial injections of adeno-associated virus encoding siBRD2 or si-NC, followed by subcutaneous injection of ISO (1.2 mg/kg/d) or an equal volume of normal saline (NS) 2 weeks after operation for 7 days. Hypertrophic changes of left ventricle were observed by **(A)** gross morphologic examination and **(B)** H&E staining, **(C)** PSR staining and **(D)** echocardiography. Scar bar: 50 μm. Post-mortem measurements of **(E)** heart weight/body weight (HW/BW) and **(F)** heart weight/tibia length (HW/TL) values. Echocardiography measurements of **(G)** ejection fraction (EF, %) and **(H)** fraction shortening (FS, %) values. **(I)** The protein expression of BRD2 and ANF measured by Western blot. The data are presented as mean ± SEM, *p < 0.05, **p < 0.01, ***p < 0.001 *vs*. control. ****p < 0.0001 *vs.* control. ^#^ *p* < 0.05, ^##^ *p* < 0.01, ^###^ *p* < 0.001 *vs.* ISO, ^###^ *p* < 0.001 *vs.* ISO, ^####^ *p* < 0.0001 *vs.* vs. ISO. *n* = 7.

We further evaluated the potential effect of BRD2 knockdown in rats with pathological cardiac hypertrophy. SD rats received intramyocardial injection of adeno-associated virus (AAV) encoding siBRD2, with NC as control, followed by subcutaneous injection of ISO (1.2 mg/kg/d) or an equal volume of normal saline (NS) for 7 days. Rats infected with AAV-siBRD2 significantly reversed ISO-induced heart enlargement by morphological observation and H&E staining (Figures 3A,B), disorder of myocardial cell arrangement (Figure 3B), interstitial fibrosis by PSR staining (Figure 3C), thickening of the left ventricle (LV) wall detected by ECG (Figure 3D), increase of heart weight/body weight (HW/BW, Figure 3E) and heart weight/tibia length (HW/TL, Figure 3F), as well as cardiac function dysregulation which is characterized by increase of ejection fraction (EF, Figure 3G) and fractional shortening (FS, Figure 3H). In addition, BRD2 knockdown suppressed ISO-induced upregulation of ANF (Figure 3I). Therefore, these *in vivo* findings confirm that BRD2 ablation confers protection against pathological cardiac hypertrophy.

### Transcriptome Analysis Revealed That Bromodomain Containing Protein 2 Participated in the Regulation of Genes Related to Cell Metabolism

**FIGURE 4:**
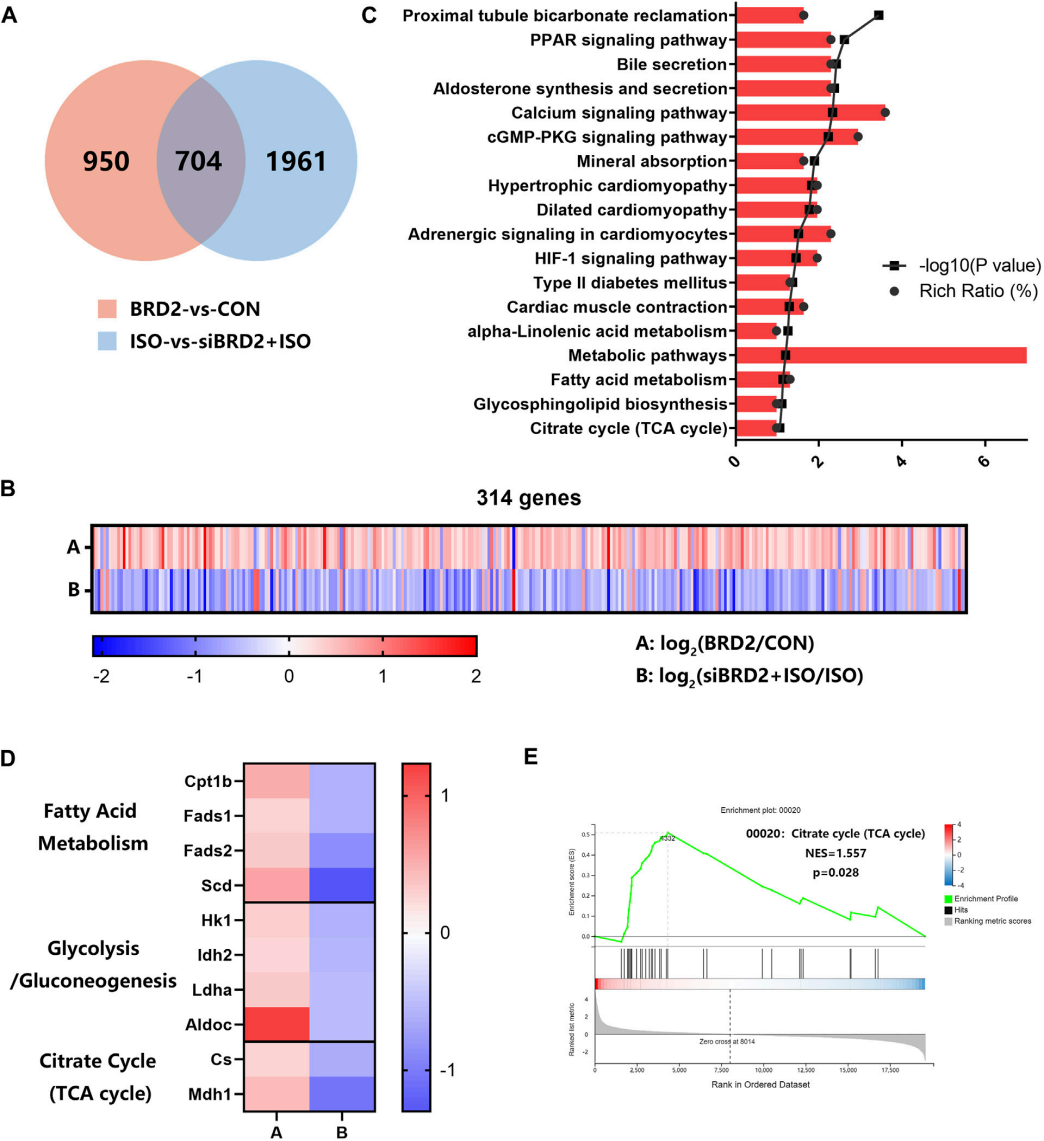
RNA sequencing analysis of transcriptome changes in NRCMs with BRD2 overexpression or silencing. **(A)** Venn diagram showing genes differentially expressed between “BRD2 vs. CON” and “siBRD2+ISO vs. ISO” groups (|log_2_FC| ≥ 0.5, Q value ≤ 0.05). **(B)** Heatmap showing differentially expressed genes between “BRD2-vs-CON” and “siBRD2+ISO” vs. “ISO” groups (|log_2_FC| ≥ 0.5, Q value ≤ 0.05). **(C)** KEGG pathway analysis of 314 genes regulated by BRD2 (|log_2_FC| ≥ 0.5, Q value ≤ 0.05). **(D)** Heatmap showing the differentially expressed metabolic genes under regulation by BRD2 according to the RNA-seq data (|log_2_FC| ≥ 0.5, Q value ≤ 0.05). **(E)** GSEA analysis of differentially expressed genes between “BRD2″ vs. “CON” group.

To explore the possible mechanisms of BRD2 in regulation of cardiac hypertrophy, we conducted RNA sequencing analysis to investigate the transcriptome changes in NRCMs with BRD2 overexpression or silencing, in the presence or absence of ISO. Using Q value (adjusted P value) ≤ 0.05 and |log2 fold change (FC)|>0.5 as the cutoff values, totally 1654 genes are differentially expressed between BRD2 overexpression and control group, while 2665 genes are differentially expressed between siBRD2+ISO and ISO group (Figure 4A). In these two gene sets, 704 genes were overlapped (Figure 4A). Among these genes, 314 genes showed opposite expression tendency in the above two gene sets, with 281 elevated in “BRD2-vs-CON” while decreased in “siBRD2+ISO-vs-ISO,” and 33 decreased in “BRD2-vs-CON” while increased in “siBRD2+ISO-vs-ISO” (Figure 4B). The KEGG pathway enrichment analysis of these 314 genes found that metabolic pathways, including fatty acid metabolism, glycosphingolipid biosynthesis and citrate cycle (TCA cycle) were part of the most important enriched pathways (Figure 4C). Heatmaps of RNA-seq data demonstrated that a variety of genes associated with fatty acid metabolism, glycolysis/gluconeogenesis or TCA cycle were upregulated in “BRD2-vs-CON,” but downregulated in “siBRD2+ISO-vs-ISO” (Figure 4D). GSEA (Gene Set Enrichment Analysis) revealed that hub genes differentially expressed between BRD2 overexpression and control were mainly enriched in “TCA cycle” (Figure 4E). These data thus hint that BRD2 might participate in the regulation of cardiomyocyte energy metabolism, in particular TCA cycle.

### Bromodomain Containing Protein 2 Modulated TCA Cycle Genes and Energy Production Process and Thus Contributed to ISO-Induced Cardiac Hypertrophy

**FIGURE 5:**
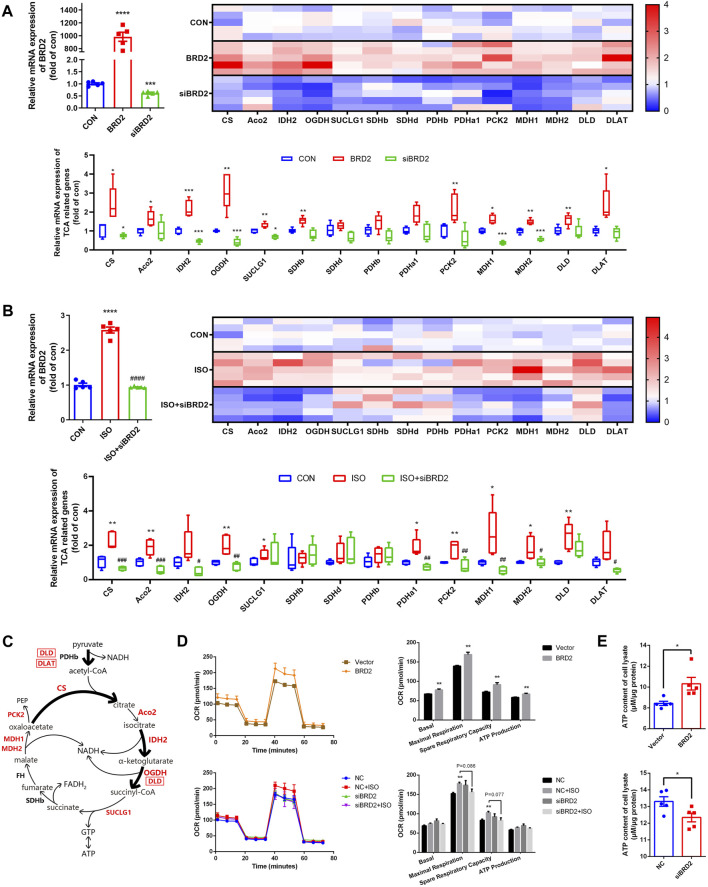
BRD2 modulated expression of TCA cycle genes and energy production during ISO-induced cardiac hypertrophy. qPCR measurement of TCA cycle genes in **(A)** NRCMs with BRD2 overexpression or knockdown, and **(B)** NRCMs with or without BRD2 knockdown or ISO treatment. **(C)** Schematic diagram showing TCA genes changes following ISO treatment in NRCMs. **(D)** Oxygen consumption rates (OCR) measured by XF Cell Mito Stress Test Kit. **(E)** ATP production in cardiomyocytes with BRD2 overexpression or knockdown. The data are presented as mean ± SEM, *p < 0.05, **p < 0.01, ***p < 0.001 *vs.* control, ****p < 0.0001 *vs.* control. ^#^ *p* < 0.05, ^##^ *p* < 0.01, ^###^ *p* < 0.001 *vs.* ISO, ^####^ *p* < 0.0001 *vs.* ISO. *n* = 5.

To further validate whether or not BRD2 is a key regulator of TCA cycle in cardiomyocytes, we measured the expressions of TCA cycle genes. Firstly, the gene expressions of TCA cycle proteins were compared between cardiomyocytes transfected with BRD2 plasmid or BRD2 siRNA. As shown in Figure 5A, a majority of TCA cycle genes, such as citrate synthase (CS), aconitase 2 (Aco2), isocitrate dehydrogenase 2 (IDH2), oxoglutarate dehydrogenase (OGDH), succinyl CoA synthetase SUCLG1, succinate dehydrogenase SDHb, phosphoenolpyruvate carboxykinase 2 (PCK2), malate dehydrogenases MDH1 and MDH2, dihydrolipoamide dehydrogenase (DLD), and dihydrolipoamide S-Acetyltransferase (DLAT), were significantly upregulated by BRD2 overexpression, but were downregulated by BRD2 silencing. Secondly, the expressions of these TCA cycle genes were studied in ISO-treated hypertrophic cardiomyocytes with or without BRD2 knockdown. The results demonstrated that the expressions of the above TCA cycle genes were augmented following ISO treatment, but were suppressed when BRD2 was knocked down (Figure 5B). Therefore, these results suggest that BRD2 is involved in the regulation of genes covered important and irreversible reactions in TCA cycle, as shown in the schematic (Figure 5C). Furthermore, the mitochondrial oxygen consumption rate (OCR) was evaluated to confirm the effect of BRD2 in the regulation of cardiac energy metabolism. Upregulation of BRD2 induced elevation of OCR, whereas BRD2 deficiency prevented ISO-induced elevation of OCR (Figure 5D). In line with these observations, BRD2 overexpression promoted ATP production while BRD2 knockdown restrained ATP production (Figure 5E). Taken in conjunction, these results suggest that BRD2 facilitates the increased energy production in response to ISO stimulation, leading to enlargement of cardiomyocytes and cardiac remodeling. Repression or inhibition of BRD2 might help to ameliorate these processes and prevent the development of cardiac hypertrophy.

## Discussion

Epigenetic “reader” BETs recognize acetylated lysine residues and recruit transcriptional complexes to initiate target gene transcription, so that exhibit critical importance in the regulation of cellular events (Gyuris et al., 2009; Belkina and Denis 2012; Bhola et al., 2021; Wang et al., 2021). We and others have reported that inhibition of BETs using pharmacological inhibitors like JQ-1 could protect against a variety of cardiovascular diseases, such as cardiac hypertrophy, atherosclerosis, angiogenesis, intimal hyperplasia, and pulmonary arterial hypertension (Spiltoir et al., 2013; Stratton and McKinsey 2015; Borck et al., 2020). Most of these studies have revealed that BRD4 is a key regulator contributing to the development of cardiovascular diseases. However, the role of other BET family members is not fully understood. In this study, we revealed that BRD2 exerted pro-hypertrophic effects via regulating cardiac energy metabolism, thus expanding our understanding of BETs in cardiovascular diseases.

The conclusion that BRD2 has a detrimental role in exacerbating pathological cardiac hypertrophy is based on the following observations: 1) expression of BRD2 was upregulated in ISO-induced pathological cardiac hypertrophy both *in vivo* and *in vitro*; 2) overexpression of BRD2 facilitated the pathogenesis of cardiomyocyte hypertrophy; 3) knockdown of BRD2 prevented ISO-induced cardiomyocyte hypertrophy; and 4) rats infected with AAV-siBRD2 ameliorated ISO-induced cardiac hypertrophy, cardiac fibrosis, and dysregulation of cardiac function. These findings thus suggest that inhibition of BRD2 is a potential strategy for treating pathological cardiac hypertrophy, and that the therapeutic potential of pan-BETi in cardiovascular diseases is at least partially attributed to BRD2 inhibition.

Mechanistically, the pro-hypertrophic effects of BRD2 are probably due to elevation of cardiac energy metabolism, taken into considerations that a series of metabolic genes were upregulated by BRD2 in the cardiomyocytes. Since the heart has a perpetually high energy demand to sustain contractile function, cardiac metabolic remodeling, referred to the disturbed substrate metabolism and abnormal mitochondrial function, is a hallmark of cardiac hypertrophy and heart failure (Lopaschuk et al., 2010; Lai et al., 2014). In most cases, the disturbance of cardiac metabolism is associated with abnormal expression and/or activity of metabolic enzymes during the pathological development of cardiac hypertrophy (van Bilsen et al., 2004; Liu et al., 2016). Indeed, our data revealed that the mRNA levels of a majority of metabolic genes, in particular those involved in TCA cycle, were augmented in ISO-induced cardiac hypertrophy, which is most likely at the compensated stage of cardiac hypertrophy since EF was increased and the basal ventricular systolic function was preserved (Osadchii 2007). These observations are in line with previous reports that ISO treatment increased the levels of TCA cycle intermediates citrate, cis-aconitate and glutamine (Peterson et al., 2021), and that mitochondria citrate formation through PDH and citrate release were both increased in the hearts of 15 week-old spontaneously hypertensive rats (SHR) (Vincent et al., 2003; Dodd et al., 2012). However, the increased expression of TCA cycle genes and improved energy production in the compensated phase could not retard the development of cardiac hypertrophy, but rather increases burden of the heart and leads to cardiac overload, eventually progressing to the decompensated stage and resulting to irreversible heart failure. The bioinformatic analysis of whole-genome sequence data demonstrated that the pro-hypertrophic effects of BRD2 was primarily due to upregulation of metabolic genes, including those involved in fatty acid metabolism, glucose metabolism and TCA cycle. By contrast, BRD2 knockdown abolished ISO-induced upregulation of those metabolic genes. Particularly, genes involved in the regulation of TCA cycle, the major downstream of fatty acid metabolism and glucose metabolism of the heart for substrate utilization and ATP production (van Bilsen et al., 2004), were identified to be important downstream target genes of BRD2. In fact, our findings about the regulatory role of BRD2 in expressions of metabolic enzymes are in line with several studies. BRD2 participates in fatty acid metabolism via altering the expression of acetyl-CoA carboxylases-α gene (ACACA), fatty acid synthase (FAS) and hormone sensitive lipase (HSL) (Deeney et al., 2016; Sun et al., 2017), in glucose metabolism via activating phosphofructokinase PFKP and enforcing Warburg effect (Xu et al., 2021), as well as in cholesterol deprivation *via* occupying the gene promoter of sigma-2 receptor (S2R) with SREBP2 (Shen et al., 2021). Consistent with results of OCR and ATP production, these findings confirm that BRD2 facilitates cardiac hypertrophy via regulating energy metabolism.

It remains unclear how BRD2 regulates the expression of metabolic genes during cardiac hypertrophy. As an important member of BET family, BRD2 can initiate the transcriptional activation process together with BRD3, by promoting RNA Pol II elongation in transcription through their histone chaperone activities that are attributed to interaction between bromodomains and acetylated chromatin (Taniguchi 2016). Thus, it is possible that BRD2 might act as a transcriptional co-activator to modulate the expression of metabolic genes. Indeed, BRD2 could interact with several transcription factors such as SREBP2, Yin Yang 1, PPAR-γ and C/EBPα, to co-occupy a majority of gene-regulatory elements and coactivate downstream target metabolic genes (Goupille et al., 2016; Bagchi et al., 2018; Shen et al., 2021; Xu et al., 2021). For this reason, BRD2 has attracted increasing attention for its critical role in regulation of cellular metabolism and metabolic diseases (Wang et al., 2009; Deeney et al., 2016; Zong et al., 2019).

Notably, BRD2 shares close similarity in structure and physiological role with BRD4 (Devaiah et al., 2016), which is well-accepted to be a nodal regulator of cardiac hypertrophy (Van der Feen et al., 2019; Zhu et al., 2020; Li et al., 2021). However, BRD4 possesses a longer CTD with a unique function that could facilitate the recruitment of P-TEFb complex to promote transcriptional elongation of hypertrophic genes, finally contributing to cardiac hypertrophy (Belkina and Denis 2012; Spiltoir et al., 2013). Thus, the lack of long CTD in BRD2 might exclude the possibility that BRD2 regulates cardiac hypertrophy in a similar mechanism as BRD4. In fact, growing evidences have illustrated that BET family members coordinate with each other in a hierarchical manner rather than simply acting in a redundant manner (Kim et al., 2021). For example, BRD2 and BRD3 coordinate to function in the initiated stage of transcriptional activation, while BRD4 functions in a later stage of transcription such as Pol II elongation (Kemp et al., 2001); BRD2 has the highest percentage of peaks at intronic and inter-genic regions, while BRD4 has the highest percentage of peaks at promoter regions (Zheng et al., 2021); loss of BRD2 reduces enhancer activity while BRD4 ablation enhances transcriptional pausing (Zheng et al., 2021); depletion of BRD2 or BRD3 results in transcriptional changes that are distinct from those caused by BRD4 depletion (Werner et al., 2020). For metabolic genes, a previous report has demonstrated that inhibition of BRD2, but not BRD4, increases fatty acid oxidation (Deeney et al., 2016). Therefore, the present findings that BRD2 regulates cardiac metabolism might elucidate a unique mechanism of BET family in the regulation of cardiac hypertrophy, in addition to that of BRD4 with the function of long CTD. However, the present data did not allow to speculate whether the regulatory mechanism of BRD2 and BRD4 in cardiac hypertrophy are connected or not.

Intriguingly, the roles of BET family members in different cardiac hypertrophic models remain to be controversial. In NRCMs stimulated by α1 receptor agonist phenylephrine or in mice induced by transverse aortic constriction (TAC), the expressions of all the BET family members remain unchanged (Anand et al., 2013) or with BRD4 upregulated only (Spiltoir et al., 2013), inconsistent with our observations that both BRD2 and BRD4 were upregulated in ISO-induced cardiac hypertrophy. Additionally, the present findings do not support the observations in a previous study showing that the hypertrophic effect of BET family were mediated by BRD4 but not BRD2 and/or BRD3 in response to phenylephrine or endothelin-1 (Martin et al., 2020). It is still unclear the exact reason leading to the discrepancy between these findings. Probably, these controversial results are due to the differences of pro-hypertrophic signaling activated by different stimuli.

Currently, a number of BETi are undergoing clinical research and most of them, including JQ-1, ABBV-075, and OTX015, target at bromodomains (Borck et al., 2020; Gokani and Bhatt 2021). By binding at the bromodomains, whether single or both, BETi displace BETs from its location on histone tails, disrupting the assembled transcriptional scaffolding and blocking expression of BET-controlled target genes (Borck et al., 2020). Owing to the conservation of bromodomains, the classic BETi could hardly suppress BETs with subtype specificity. The present results that BRD2 exerted pro-hypertrophic effects in cardiomyocytes, apart from the previously reported BRD4, might help to explain the promising effect of pan-BETi in treatment of pathological cardiac hypertrophy, rather than specific inhibitor of a single BET member.

In conclusion, the present study identified BRD2 as a novel regulator of pathological cardiac hypertrophy, which could facilitate cardiac hypertrophy through upregulating gene expression of cardiac metabolic enzymes in particular those in TCA cycle. Strategies targeting inhibition of BRD2 might suggest therapeutic potential for pathological cardiac hypertrophy and heart failure.

## Data Availability

The datasets presented in this study can be found in online repositories. The names of the repository/repositories and accession number(s) can be found below: https://www.ncbi.nlm.nih.gov/bioproject/, PRJNA830755.
