# Supplementary material for: Epigenetic Reader Bromodomain Containing Protein 2 Facilitates Pathological Cardiac Hypertrophy via Regulating the Expression of Citrate Cycle Genes
Source: Front Pharmacol. 2022 May 25;13:887991. doi: 10.3389/fphar.2022.887991 (PMC9174549; doi:10.3389/fphar.2022.887991)
Supplement: Supplementary file 1 [file DataSheet1.docx]

Supplementary Material

# Supplementary Figures and Tables

## 1.1 Supplementary Figures


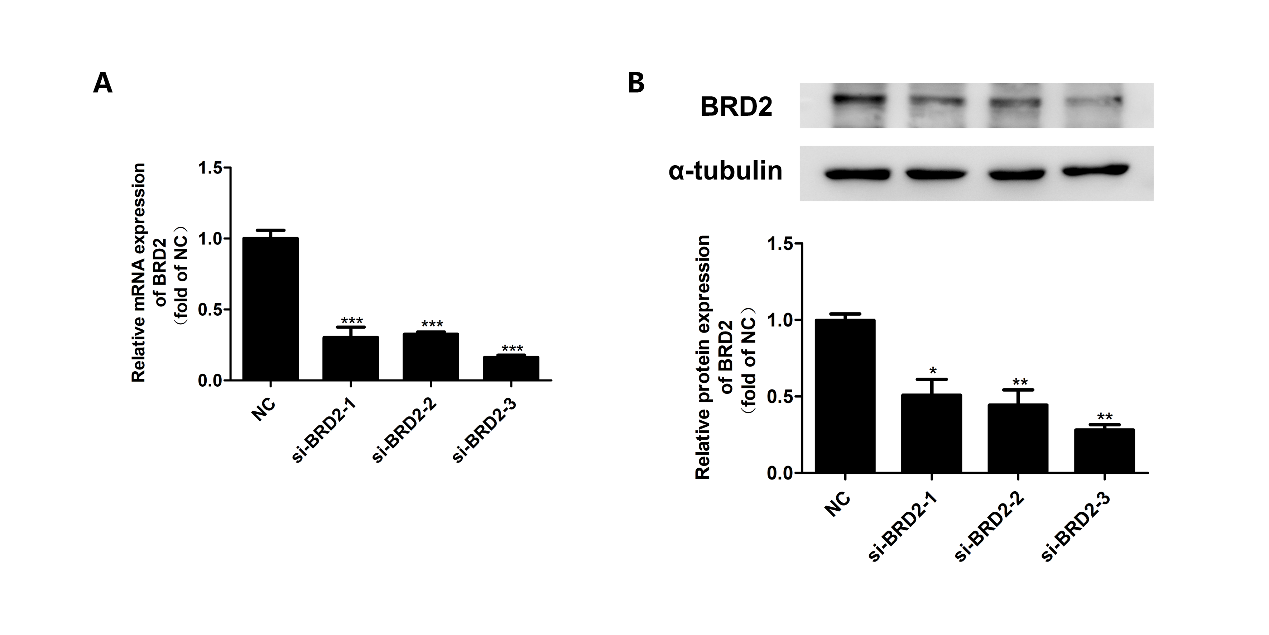


**Supplementary Figure S1. (A)** The mRNA level of BRD2 after transfected with 3 siRNA targeting BRD2 (siBRD2) or negative control (NC) for 48hr. **(B)** The protein expression of BRD2 after transfected with 3 siRNA targeting BRD2 (siBRD2) or negative control (NC) for 48hr. The data are presented as mean±SEM, **P*<0.05, ***P*<0.01, ****P*<0.001 *vs.* control. *n*=3.

## 1.2 Supplementary Tables

**Supplementary Table S1**

The primer sequences used for qPCR.

| **Primer** | **Sense (5’-3’)** | **Anti-sense (5’-3’)** |
| --- | --- | --- |
| β-Actin | TCGTGCGTGACATTAAAGAG | CCGTATACAGTGCGGTGTCC |
| BRD2 | AGCACTGTCAAGCGGAAGAT | GGCAAGGCAGTAGAGACAGG |
| ANF | CCGTATACAGTGCGGTGTCC | CAGAGAGGGAGCTAAGTGCC |
| BNP | AGCTGCTTTGGGCAGAAGAT | AAAACAACCTCAGCCCGTCA |
| CS | GAACTCATCCTGCCTCGTCCTTG | CTGTCTTCCCATGCTGCTGTCTG |
| Aco2 | AGGTGGGTGGTGATTGGAGATGAG | TTGCGAAGCTCTTGGTGATGATGG |
| IDH2 | GTGGAGATGGATGGCGATGAGATG | ATTGGTCTGGTCACGGTTTGGAAG |
| OGDH | CCGTGCCCGCTGACATTATCTC | CCGATGAAAGTGGTGGTGGGTAAG |
| SUCLG1 | TCCCTCCTCCTTTCGCTGCTG | CACCATGTCCTGCTGCGGAATAC |
| SDHb | CCGATGGTGCTGGATGCTCTAATC | AGCGTGTTGCCTCCGTTGATG |
| SDHd | CAGCACATCCACCTGTCACCAAG | CCACCACAGAGCAGGGATTCAAG |
| PDHb | GACCTTTGCGGCAGGCTTCC | TGGCTTCACGAACTGTCAACTGC |
| PDHa1 | ACGAAGAGGAGGCTGTGCTAAGG | CGATACCGTTGCCGCCATAGAAG |
| PCK2 | CAGGCTGGAAAGTGGAGTGTGTG | CATTGGGATTGGTGGTGGCAGAG |
| MDH1 | GGGAGGGCATGGAGAGGAAGG | AGGCAGTTTGTATTGGCTGGGTTC |
| MDH2 | TCAGCACTTCAGCCCAGAACAATG | CCAGGTGTGTGAGCGATGTCATAG |
| DLD | ACGGCCCTTTACGCAGAATTTGG | ACATTGGACCAGCAACCACATCTC |
| DLAT | AGACATCCCCATCAGCAACATTCG | CTTCCGCACCAACAGCACCTC |
